# Associations Between Avoidant/Restrictive Food Intake Disorder Dimensions and Obsessive‐Compulsive Symptomatology

**DOI:** 10.1002/erv.70122

**Published:** 2026-04-26

**Authors:** Mariana Valdez Aguilar, Gabriela K. Giulumian, Isabel Rodriguez, Jessica H. Baker, Preina P. Surti, Lisa Dinkler, Jerry Guintivano, Jessica S. Johnson, Casey M. MacDermod, Shelby N. Ortiz, Emily M. Pisetsky, Jennifer P. White, Nadia Micali, Cynthia M. Bulik, Laura M. Thornton, Ya‐Ke Wu

**Affiliations:** ^1^ Department of Psychiatry University of North Carolina at Chapel Hill Chapel Hill North Carolina USA; ^2^ Department of Psychology and Neuroscience University of North Carolina at Chapel Hill Chapel Hill North Carolina USA; ^3^ School of Medicine University of North Carolina at Chapel Hill Chapel Hill North Carolina USA; ^4^ Department of Nutrition University of North Carolina at Chapel Hill Chapel Hill North Carolina USA; ^5^ Department of Medical Epidemiology and Biostatistics Karolinska Institutet Stockholm Sweden; ^6^ Center for Eating and Feeding Disorders Research Mental Health Center Ballerup Copenhagen University Hospital—Mental Health Services CPH Copenhagen Denmark; ^7^ Great Ormond Street Institute of Child Health University College London London UK; ^8^ Institute for Biological Psychiatry Mental Health Centre Sct Hans Copenhagen University Hospital—Mental Health Services Copenhagen Denmark; ^9^ School of Nursing University of North Carolina at Chapel Hill Chapel Hill North Carolina USA

**Keywords:** anxiety, depression, eating disorders, feeding disorders, obsessive‐compulsive symptomatology

## Abstract

**Objective:**

Although obsessive‐compulsive disorder (OCD) commonly co‐occurs with anorexia nervosa and bulimia nervosa, less is known about its relationship with avoidant/restrictive food intake disorder (ARFID) dimensions. Whether associations between ARFID dimensions and OCD differ by sex is also unclear. We examined associations between ARFID dimensions and OCD symptoms by sex, accounting for depression and anxiety.

**Methods:**

Data from 3120 participants ages 15+ in the ARFID Genes and Environment (ARFID‐GEN) study were analyzed. ARFID dimensions (i.e., sensory sensitivity; low appetite/lack of interest in food; and fear of aversive consequences), OCD symptoms, anxiety, and depression were assessed using validated self‐report measures. We evaluated associations between ARFID dimensions and OCD symptoms using general linear models, adjusting for depression and anxiety.

**Results:**

ARFID dimensions were significantly associated with OCD symptoms. After adjusting for depression and anxiety, these associations were attenuated but remained significant for all ARFID dimensions. Associations between ARFID dimensions and OCD symptoms varied by sex.

**Conclusion:**

The relation between ARFID symptomatology and OCD warrants further exploration across the developmental spectrum in which ARFID appears. Clinically, bidirectional screening may improve diagnostic clarity and be informative to tailor interventions appropriately.

**Trial Registration:**

ARFID‐GEN is registered in clinicaltrials.gov (NCT05605067)

## Highlights

- We conducted the ARFID Genes and Environment (ARFID‐GEN) study, the largest known study of its kind including 3120 participants ages 15+.
- Significant positive associations emerged between all ARFID dimensions and OCD symptoms even after adjusting for depression and anxiety and while including age and sex as covariates.
- ARFID dimensions and OCD symptoms are related.
- Clinically, bidirectional screening may be informative to tailor interventions appropriately.

## 1 Introduction

Avoidant/restrictive food intake disorder (ARFID) involves limited food intake leading to significant weight loss, nutritional deficiency, or psychosocial impairment and includes three non‐mutually exclusive phenotypes: (1) sensory sensitivity; (2) low appetite/lack of interest in food; and (3) fear of aversive consequences (American Psychiatric Association 2013). Given symptom similarities–such as food neophobia and intrusive thoughts about aversive consequences (Kinnaird et al. 2017; Norris et al. 2014; Zickgraf et al. 2016)–we explored associations between ARFID dimensions and obsessive‐compulsive disorder (OCD) symptoms.

OCD commonly co‐occurs with anorexia nervosa (∼20%‐25%), bulimia nervosa (∼12%‐15%), and binge‐eating disorder (∼5%) [for example (Drakes et al. 2021; Kapadia et al. 2025; Kaye et al. 2004; Mandelli et al. 2020). Twin and molecular genetic studies suggest that OCD and anorexia nervosa share some genetic factors (The Brainstorm Consortium et al. 2018; Cederlof et al. 2015; Termorshuizen et al. 2025; Watson et al. 2019; Xu et al. 2019; Yilmaz et al. 2020). Similar patterns may exist for ARFID, as empirical evidence supports an association between ARFID and OCD. In youth with threshold or subthreshold ARFID (Kambanis et al. 2020), only 4% met criteria for OCD, however comorbidity within the anxious/obsessive/trauma domain was associated with higher scores on sensory sensitivity and fear of aversive consequences. Lieberman et al. (2019) found 21% of treated children with ARFID had OCD. In a Swedish cohort, children with ARFID had increased risk of parent‐reported OCD (Nyholmer et al. 2025), though no increase in clinically diagnosed OCD was found in health register data (Wronski et al. 2025). Less is known about ARFID‐OCD co‐occurrence in adults, although adults with picky eating (Kauer et al. 2015) or ARFID (Zickgraf et al. 2016) score higher on obsessive‐compulsive measures than non‐picky eaters.

Research suggests that ARFID dimensions differ in their associations with OCD. Sensory sensitivity and fear of aversive consequences have been linked to anxious/obsessive traits (Kambanis et al. 2020), rigid avoidance behaviors, and persistent preoccupations (Norris et al. 2014). A selective eating study supports the role of sensory sensitivity and cognitive rigidity in ARFID‐like symptoms (Zickgraf et al. 2022), and shows that picky eating with ARFID symptoms is associated with greater internalizing distress and OCD symptoms than picky eating alone (Zickgraf et al. 2016). Thus, we hypothesized that OCD symptoms would be more strongly related to sensory‐based avoidance and fear of aversive consequences than low appetite. However, it is unclear how depression and anxiety may impact these associations given that they occur with ARFID (Sanchez‐Cerezo et al. 2023) and OCD (Quarantini et al. 2011). Further, OCD has strong and positive genetic correlations with anxiety and depression (Strom et al. 2024).

Sex differences have been observed in both disorders. Men with OCD more often report sexual and religious obsessions; women report more contamination‐related concerns (Mathes et al. 2019; Torresan et al. 2013). Findings on sex differences in ARFID are mixed–one study report no differences (Zickgraf et al. 2019), another suggests men report more sensory sensitivity (Katzman et al. 2021). These inconsistencies highlight the need for research into sex‐specific associations.

This study examined associations between ARFID dimensions and OCD symptoms in a large sample of adults and adolescents, accounting for depression and anxiety. We hypothesized: (1) significant associations between ARFID dimensions and OCD symptoms; and (2) partial attenuation after adjusting for depression and anxiety.

## 2 Methods

### 2.1 Study Design

We report secondary analyses from the Avoidant Restrictive Food Intake Disorder‐Genes and Environment (ARFID‐GEN) study, a cross‐sectional investigation examining genetic and environmental risk factors for ARFID (Bulik et al. 2023). ARFID‐GEN is registered in clinicaltrials.gov (NCT05605067).

### 2.2 Participants

Participants aged 15+ met threshold scores (Burton Murray et al. 2021) on at least one dimension of the Nine Item Avoidant/Restrictive Food Intake Disorder Screen [NIAS (Zickgraf and Ellis 2018);] and endorsed at least one criterion A feature on the Pica, ARFID & Rumination Disorder Inventory Questionnaire [PARDI‐AR‐Q (Bryant‐Waugh et al. 2022);], per proposed ARFID diagnostic criteria (Zickgraf et al. 2025). Inclusion required a global score < 4 on the Eating Disorder Examination Questionnaire (Fairburn and Beglin 1994), no vomiting or laxative use, and ≤ 4 episodes of binge eating in the last 28 days (total excluded = 1395). These extended criteria minimized overlap with other eating disorders.

### 2.3 Measures

#### 2.3.1 Demographic Variables

Participants reported age and sex at birth. Data for current height and weight were collected from the PARDI‐AR‐Q to calculate BMI.

#### 2.3.2 ARFID Dimensions

The NIAS uses nine items, with scores ranging from 0 (strongly disagree) to 5 (strongly agree), to self‐report ARFID‐associated eating behaviors (Zickgraf and Ellis 2018). It captures three dimensions: *picky eating* (sensory aversion to food); *low appetite* (lack of interest in eating or food); and *fear* (fear of aversive consequences associated with eating), which are calculated as the sum of the respective items. Cronbach's alphas for the dimensions ranged from 0.82–0.89.

The PARDI‐AR‐Q (Bryant‐Waugh et al. 2022) uses nine items to assess three ARFID dimensions: *sensory‐based avoidance* (sensory aversion to food); *lack of interest* (lack of interest in eating or food); and *concern about aversive consequences* (fear of aversive consequences associated with eating). Dimensions are mean scores of component items, with response options ranging from 0 (e.g., no/never) to 6 (e.g., extremely/always). Cronbach's alphas for dimensions ranged from 0.75 to 0.87.

#### 2.3.3 OCD Symptoms

The 18‐item Obsessive‐Compulsive Inventory–Revised OCI‐R (Foa et al. 2002), was completed by ∼86% of participants. Items are scored using a 5‐point Likert scale and are summed for a total score (*OCI‐R total*; range: 0–72). Subscales were scored according to published criteria. Cronbach's alpha for OCI‐R total was 0.83.

#### 2.3.4 Depression Symptoms

Depression (range: 0–24) was assessed using the sum of the first eight items of Patient Health Questionnaire‐9 [PHQ‐9 (Kroenke et al. 2001)] in adults and the sum of the first eight items of PHQ modified for Adolescents [PHQ‐A (Kroenke et al. 2009)] for participants aged 15–17. Item 9 (suicidal ideation) was not assessed in adolescents so was not included in the score. Both questionnaires use a 4‐point Likert scale to measure severity of current depressive symptoms. Cronbach's alphas were 0.87 (adults) and 0.82 (adolescents).

#### 2.3.5 Anxiety Symptoms

Anxiety (range: 0–21) over the past 2 weeks was assessed with the General Anxiety Disorder‐7 (Mossman et al. 2017). Symptom frequency is rated on a 4‐point Likert scale. The anxiety score is calculated by summing the items. Cronbach's alpha was 0.90.

### 2.4 Data Analysis

Data were analyzed with SAS 9.4 (SAS Institute Inc 2004). Of 3800 initial participants, missing data for OCI‐R total, depression, and anxiety ranged from 0.06% to 1.92%. No imputation was applied since all variables had < 5% missing data (Graham 2009). In total, 680 (18%) participants were excluded because the OCI‐R total was not completed or data were missing for scales relevant to the analysis. The analytic sample comprised 3120 participants: (3073 adults, 47 adolescents) and was compared to the excluded sample on demographic variables and ARFID dimensions. Samples differed only in age (the analytic group was younger; Cohen's *d* = 0.24).

Descriptive statistics and Pearson's correlations were computed. General linear models (GLMs) examined sex differences on ARFID dimensions, OCI‐R total, depression, and anxiety.

GLMs were applied to evaluate associations between ARFID dimensions (independent variables) and OCI‐R total (dependent variable) and the impact of depression and anxiety on those associations. A threshold of *r* = 0.10, a small correlation (Gignac and Szodorai 2016), was set as the minimum criterion for evaluating associations between ARFID dimensions and OCI‐R total. Thus, NIAS‐picky eating was excluded from evaluation (*r* = 0.08). In each model, only one ARFID dimension was included as an independent variable. Model 1 evaluated associations between each ARFID dimension and OCI‐R total. In Model 2, depression was added to Model 1 analyses to evaluate its effect on respective associations. In Model 3, anxiety was added to Model 1 analyses to evaluate its effect on respective associations. Model 4 added both depression and anxiety to Model 1 analyses to evaluate their joint effect on respective associations. Given the age range of the sample, mixed findings on sex differences in ARFID and OCD prevalence (Dinkler et al. 2023, 2022; Farag et al. 2022; Hilbert et al. 2021; Mataix‐Cols et al. 2013; Mathes et al. 2019; Nitsch et al. 2023), and that ARFID and OCD symptom expression varies between the sexes (Katzman et al. 2021; Mathes et al. 2019; Torresan et al. 2013), we included age and sex as covariates in all models. If sex was significant in any Model 1 association, the respective analysis was stratified by sex. As a secondary analysis, all models were applied to each OCI‐R subscale (full subscale results are in supplementary materials). Multiple testing was corrected using False Discovery Rate (Benjamini and Hochberg 1995), *q*‐values (the corrected *p*‐values) are presented.

## 3 Results

### 3.1 Participant Characteristics: Descriptive and Correlation Results

Descriptive statistics are shown in Table 1. The mean (SD) age of the total sample was 30.7 (12.6) years and the mean BMI was 26.7 (8.4) kg/m^2^. Our sample was primarily female (91.2%), White (86.2%), and non‐Hispanic (88.8%).

**TABLE 1:** Demographic characteristics of participants for the total sample (*n* = 3120).

Characteristics		Mean (SD)
Age (years) (range = 15–83)		30.7 (12.6)
BMI (kg/m^2^) (range = 8.7–93.3)		26.7 (8.4)
		*N* (%)
Sex	Male	274 (8.8)
	Female	2846 (91.2)
Race	White	2690 (86.2)
	Black	103 (3.3)
	Asian	73 (2.3)
	Pacific Islander	NR
	Native American	NR
	More than one race	173 (5.5)
	Prefer not to answer	41 (1.3)
Ethnicity	Hispanic	246 (7.9)
	Non‐Hispanic	2768 (88.8)
	Prefer not to answer	105 (3.4)

Abbreviations: SD, standard deviation; NR, cells with less than 2% of the total are not reported to protect the privacy of participants.

Table 2 presents Pearson correlations between NIAS and PARDI‐AR‐Q dimensions, OCI‐R total, depression, and anxiety. OCI‐R total was significantly positively correlated with all ARFID dimensions. Most ARFID dimensions were significantly correlated with depression and anxiety. OCI‐R total, depression, and anxiety were significantly correlated with each other.

**TABLE 2:** Pearson correlations between NIAS and PARDI‐AR‐Q dimensions with OCI‐R total, depression, and anxiety.

	NIAS			PARDI‐AR‐Q			OCI‐R Total	Depression	Anxiety
	Picky eating	Low appetite	Fear	Sensory‐based avoidance	Lack of interest	Concern about aversive consequences			
NIAS									
Picky eating	1	−0.12^a^	−0.09^a^	0.48^a^	−0.01	0.03	0.08^a^	0.03	0.04^a^
Low appetite		1	0.33^a^	0.11^a^	0.71^a^	0.20^a^	0.15^a^	0.22^a^	0.15^a^
Fear			1	0.12^a^	0.29^a^	0.71^a^	0.20^a^	0.13^a^	0.18^a^
PARDI‐AR‐Q									
Sensory‐based avoidance				1	0.28*	0.24^a^	0.28^a^	0.18^a^	0.20^a^
Lack of interest					1	0.27^a^	0.23^a^	0.31^a^	0.25^a^
Concern about aversive consequences						1	0.24^a^	0.15^a^	0.24^a^
OCI‐R total							1	0.46^a^	0.56^a^
Depression (PHQ‐8)								1	0.66^a^
Anxiety (GAD‐7)									1

*Note:* Effect sizes of correlations can be interpreted as follows: Small = 0.10, Medium = 0.30, Large = 0.50.

Abbreviations: NIAS, Nine Item Avoidant/Restrictive Food Intake Disorder Screen; OCI‐R, total score from the Obsessive‐Compulsive Inventory‐Revised; PARDI‐AR‐Q, Pica, ARFID, & Rumination Disorder Inventory Questionnaire; SD, standard deviation.

^a^ significant at the *p* < 0.05 level.

Table 3 presents mean scores for ARFID dimensions, OCI‐R total, depression, and anxiety for the total sample and by sex. ARFID dimension scores for females were significantly higher than for males for NIAS‐low appetite, PARDI‐AR‐Q‐sensory‐based avoidance, and PARDI‐AR‐Q‐lack of interest. There were no significant sex differences on NIAS‐picky eating, NIAS‐fear, and PARDI‐AR‐Q‐concern about aversive consequences. Females had significantly higher scores for OCI‐R total and anxiety than males. The OCI‐R total mean was 20.7, just below the clinical cut‐off of 21. To contextualize, 1369 (43.9%) participants had scores ≥ 21: ∼35% of participants met cutoffs for OCI‐R total and NIAS picky eating (*r*_tet_ = 0.09) and NIAS low appetite (*r*_tet_ = 0.16), ∼22% met cutoffs for OCI‐R total and NIAS fear (*r*_tet_ = 0.21).

**TABLE 3:** Mean (SD) for ARFID symptom dimensions, OCI‐R total, depression, and anxiety for the total sample and by sex. Results and effect sizes from general linear models (GLM) comparing males and females.

Variables	Total sample *n* = 3120	Male *n* = 274	Female *n* = 2846	GLM Results	Effect size
	M (SD)	M (SD)	M (SD)	F (*q*‐value)^a^	η^2^ ^b^
NIAS					
Picky eating	11.9 (3.1)	12.0 (3.1)	11.9 (3.1)	0.22 (0.70)	0.000
Low appetite	10.6 (3.8)	9.7 (4.1)	10.7 (3.7)	14.77 (0.0002)	0.005
Fear	7.9 (4.7)	7.9 (4.5)	7.9 (4.7)	0.04 (0.84)	0.000
PARDI‐AR‐Q					
Sensory‐based avoidance	3.7 (1.6)	3.3 (1.8)	3.7 (1.6)	19.07 (0.0002)	0.006
Lack of interest	4.0 (1.2)	3.6 (1.4)	4.1 (1.2)	32.28 (0.0001)	0.010
Concern about aversive consequences	2.3 (1.8)	2.4 (1.8)	2.3 (1.8)	1.53 (0.27)	0.000
OCI‐R total	20.7 (14.3)	17.4 (13.3)	21.0 (14.4)	16.72 (0.0002)	0.005
Depression	11.3 (5.8)	10.4 (6.4)	11.3 (5.7)	7.43 (0.012)	0.002
Anxiety	9.4 (5.7)	7.9 (5.6)	9.6 (5.7)	22.12 (0.0002)**	0.007

Abbreviations: ARFID, Avoidant/restrictive food intake disorder; NIAS, Nine Item Avoidant/Restrictive Food Intake Disorder Screen; PARDI‐AR‐Q, the Pica, ARFID, & Rumination Disorder Inventory Questionnaire; OCI‐R, Obsessive‐Compulsive Inventory‐Revised; SD, standard deviation.

^a^ False Discovery Rate *p*‐values (labeled as *q*‐values) are presented.

^b^ Effect size (eta squared, η^2^) is interpreted as follow: Small = 0.01, Medium = 0.06, Large = 0.14.

### 3.2 Association Between ARFID Dimensions and OCD Symptoms

Table 4 presents results from the GLMs evaluating the associations of ARFID dimensions with OCI‐R total, adjusted for age and sex. In Model 1, all ARFID dimensions were positively associated with OCI‐R total. When accounting for depression (Model 2), all associations were attenuated (regression coefficients were reduced) but remained significant. In Model 3 (accounting for anxiety), all associations were attenuated but remained significant.

**TABLE 4:** Results from general linear models examining associations between ARFID dimensions and OCI‐R total (Model 1) and when accounting for depression (Model 2), anxiety (Model 3), and both depression and anxiety (Model 4) for the total sample. Age and sex were entered into all models as covariates.

	Model 1			Model 2			Model 3			Model 4		
	*B (95% CI)*	F (*q*‐value)^a^	η_p_^2^ ^b^	*B (95% CI)*	F (*q*‐value)^a^	η_p_^2^ ^b^	*B (95% CI)*	F (*q*‐value)^a^	η_p_^2^ ^b^	*B (95% CI)*	F (*q*‐value)^a^	η_p_^2^ ^b^
NIAS												
Low appetite	0.45 (0.33, 0.59)	48.78 (0.0001)	0.01	0.13 (0.02, 0.25)	4.88 (0.028)	0.00	0.20 (0.09, 0.31)	12.94 (0.0003)	0.00	0.13 (0.02, 0.24)	5.61 (0.018)	0.00
Fear	0.64 (0.54, 0.74)	153.33 (0.0001)	0.04	0.47 (0.37, 0.56)	96.99 (0.0001)	0.03	0.35 (0.26, 0.44)	58.98 (0.0001)	0.02	0.34 (0.25, 0.43)	57.84 (0.0001)	0.02
PARDI‐AR‐Q												
Sensory‐based avoidance	2.04 (1.73, 2.35)	164.42 (0.0001)	0.05	1.47 (1.19, 1.76)	102.63 (0.0001)	0.04	1.36 (1.09, 1.62)	97.32 (0.0001)	0.03	1.28 (1.01, 1.55)	88.21 (0.0001)	0.01
Lack of interest	2.28 (1.88, 2.68)	126.65 (0.0001)	0.04	0.83 (0.46, 1.22)	18.98 (0.0001)	0.01	0.99 (0.64, 1.34)	30.74 (0.0001)	0.02	0.71 (0.36, 1.07)	15.49 (0.0001)	0.00
Concern about aversive consequences	2.05 (1.79, 2.32)	230.94 (0.0001)	0.06	1.55 (1.21, 1.79)	156.17 (0.0001)	0.05	1.04 (0.80, 1.28)	73.88 (0.0001)	0.02	1.06 (0.83, 1.30)	78.33 (0.0001)	0.02

Abbreviations: *B*, parameter estimates; NIAS, Nine Item Avoidant/Restrictive Food Intake Disorder Screen; PARDI‐AR‐Q, the Pica, ARFID, & Rumination Disorder Inventory Questionnaire.

^a^ False Discovery Rate *p*‐values (labeled as *q*‐values) are presented.

^b^ Effect size (partial eta squared; η_p_^2^) is interpreted as follow: Small = 0.01, Medium = 0.06, Large = 0.14.

Comparing Models 2 and 3, depression had a greater effect than anxiety on associations of NIAS‐low appetite and PARDI‐AR‐Q‐lack of interest with OCI‐R total. Anxiety had a greater effect than depression on the remaining associations.

When both depression and anxiety were included in the Model 4, associations between NIAS‐low appetite, NIAS‐fear, and PARDI‐AR‐Q‐concern about aversive consequences with OCI‐R total remained similar to those in Models 2 and 3. In contrast, the associations between PARDI‐AR‐Q‐lack of interest and PARDI‐AR‐Q‐sensory‐based avoidance with OCI‐R total were further attenuated compared to Model 2 or 3, although the confidence intervals overlapped. All associations remained significant in Model 4.

Sex‐stratified results are presented in Table 5. Sex was a significant factor in Model 1 for NIAS‐low appetite (*q* = 0.045), NIAS‐fear (*q* = 0.029), and PARDI‐AR‐Q‐concern about aversive consequences (*q* = 0.017). Results for females were similar to the results for the full sample, reflecting our predominantly female sample. For males, results differed somewhat. The regression coefficients in males for NIAS‐fear (*B* = 0.45) and PARDI‐AR‐Q‐concern about aversive consequences (*B* = 1.54) were smaller than in females (*B* = 0.66 *and B* = 2.11, respectively). These associations were fully attenuated by both depression and anxiety in males.

**TABLE 5:** Results from linear regression models examining associations between ARFID dimensions and OCI‐R total (Model 1) and when accounting for depression (Model 2), anxiety (Model 3), and both depression and anxiety (Model 4), stratified by sex. Age was entered into all models as a covariate.

	Model 1			Model 2			Model 3			Model 4		
	*B (95% CI)*	F (*q*‐value^a^)	η_p_^2^ ^b^	*B (95% CI)*	F (*q*‐value^a^)	η_p_^2^ ^b^	*B (95% CI)*	F (*q*‐value^a^)	η_p_^2^ ^b^	*B (95% CI*)	F (*q*‐value^a^)	η_p_^2^ ^b^
Males												
NIAS												
Low appetite	0.46 (0.08, 0.83)	5.71 (0.018)	0.02	0.02 (−0.31, 0.35)	0.01 (0.921)	0.00	0.18 (−0.14, 0.51)	1.23 (0.268)	0.00	0.04 (−0.29, 0.36)	0.05 (0.826)	0.00
Fear	0.45 (0.11, 0.80)	6.70 (0.017)	0.02	0.24 (−0.05, 0.53)	2.68 (0.155)	0.01	0.18 (−0.14, 0.46)	1.33 (0.268)	0.00	0.18 (−0.11, 0.47)	1.49 (0.335)	0.01
PARDI‐AR‐Q												
Concern about aversive consequences	1.54 (0.70, 2.39)	12.83 (0.002)	0.05	0.90 (0.17, 1.63)	5.86 (0.051)	0.02	0.74 (0.00, 1.50)	3.83 (0.156)	0.01	0.73 (0.00, 1.45)	3.93 (0.147)	0.01
Females												
NIAS												
Low appetite	0.45 (0.32, 0.59)	42.63 (0.0001)	0.01	0.14 (0.01, 0.27)	4.76 (0.030)	0.00	0.20 (0.08, 0.32)	11.43 (0.0007)	0.00	0.14 (0.02, 0.26)	5.34 (0.021)	0.00
Fear	0.66 (0.55, 0.77)	147.70 (0.0001)	0.05	0.49 (0.39, 0.59)	95.51 (0.0001)	0.03	0.37 (0.27, 0.46)	58.88 (0.0001)	0.02	0.36 (0.27, 0.45)	57.56 (0.0001)	0.02
PARDI‐AR‐Q												
Concern about aversive consequences	2.11 (1.84, 2.40)	222.47 (0.0001)	0.07	1.63 (1.36, 1.87)	154.59 (0.0001)	0.05	1.08 (0.83, 1.34)	72.03 (0.0001)	0.02	1.11 (0.86, 1.35)	76.33 (0.0001)	0.03

Abbreviations: *B*, parameter estimates; NIAS, Nine Item Avoidant/Restrictive Food Intake Disorder Screen; PARDI‐AR‐Q, the Pica, ARFID, & Rumination Disorder Inventory Questionnaire.

^a^ False Discovery Rate *p*‐values (labeled as *q*‐values) are presented.

^b^ Effect size (partial eta squared; η_p_^2^) is interpreted as follow: Small = 0.01, Medium = 0.06, Large = 0.14.

Results for the OCI‐R subscales are presented in supplemental materials (Supporting Information S1: Tables S1–S3). OCI‐R total is highly correlated with all OCI‐R subscales. Sex differences were observed in mean scores for all subscales except washing and checking. In general, patterns of associations between AFRID dimensions and OCI‐R subscales were similar to those seen for OCI‐R total.

## 4 Discussion

In a large sample of adults and adolescents with ARFID symptoms, ARFID dimensions and OCI‐R total were significantly associated. These associations were only partly attenuated after adjusting for depression and anxiety suggesting that the relationship between ARFID dimensions and OCI‐R total scores persists independently to some extent. Effect sizes were small, but even modest associations can lead to meaningful impairment, particularly when symptoms co‐occur, given that ARFID and OCD are linked to reduced quality of life, nutritional compromise, and functional impairment (Hay et al. 2017; Kinter et al. 2024).

Sex significantly influenced associations between NIAS‐low appetite, NIAS‐fear, and PARDI‐AR‐Q‐concern about aversive consequences with OCI‐R total. Sex stratification yielded similar estimates in males and females for NIAS‐low appetite and larger estimates in females for NIAS‐fear and PARDI‐AR‐Q‐concern about aversive consequences, though the small male sample (9%) limits interpretation. However, depression and anxiety partially attenuated the associations in females, which remained significant, but fully explained these associations in males.

All dimensions except NIAS‐picky eating (the least correlated with OCI‐R total) had similar correlations with the OCI‐R total (from 0.15 to 0.28). However, based on item composition, PARDI‐AR‐Q‐sensory‐based avoidance and fear of aversive consequences could have been expected to be more strongly associated with OCI‐R total as they show parallels with obsessive avoidance, compulsive behaviors, and rumination in OCD. These differential patterns highlight heterogeneity within the ARFID diagnosis and across its assessments.

Clinical samples help clarify the nature of pathology and co‐occurring conditions, whereas non‐clinical samples reveal how the disorder manifests in the broader population. ARFID prevalence in non‐clinical samples ranges from 0.3% to 15.5% (D'Adamo et al. 2023), highlighting the value of including both clinical and community participants in our recruitment.

Several limitations should be noted. First, our sample was restricted to individuals meeting clinical criteria the NIAS and PARDI‐AR‐Q. Consequently, variability in ARFID symptoms is limited, and findings may not generalize to individuals with subthreshold symptoms or to broader populations. Future research including participants below the clinical cut‐offs would help address this limitation. Second, reliance on self‐reported measures introduces potential biases that may affect data accuracy. In ARFID‐GEN, the PARDI‐AR‐Q showed better specificity than the NIAS suggesting it may be more effective in accurately identifying individuals with ARFID (Ortiz et al. 2025). Third, although our broadly‐recruited sample enhances generalizability by capturing clinical and community cases, the predominance of females (91%) limits representativeness and calls for more balanced recruitment. Further, our ability to detect associations in males may have been limited, potentially biasing results (Heidari et al. 2016). Similar limitations have been noted in prior ARFID studies (Katzman et al. 2021; Vanzhula et al. 2023). Thus, the observed sex‐specific findings should be interpreted with caution.

In conclusion, ARFID dimensions were significantly associated with OCI‐R total, underscoring the value of routine bidirectional screening: assessing OCD in individuals with ARFID to detect potentially complicating symptoms and evaluating ARFID symptoms in individuals with OCD to identify disordered eating presentations that may otherwise go unrecognized. Integrating OCD‐informed exposure strategies into ARFID treatments, including Cognitive Behavioral Therapy for ARFID (Eddy and Thomas 2019; Thomas et al. 2020) and Family‐Based Treatment (Lock 2021), may enhance their effectiveness.

## Author Contributions

**Mariana Valdez Aguilar:** conceptualization, writing – original draft, writing – review and editing. **Gabriela K. Giulumian:** conceptualization, writing – original draft, writing – review and editing. **Isabel Rodriguez:** writing – review and editing. **Jessica H. Baker:** writing – review and editing, funding acquisition. **Preina P. Surti:** conceptualization, writing – original draft, writing – review and editing. **Lisa Dinker:** writing – review and editing, supervision. **Jerry Guintivano:** software, investigation, resources, data curation, writing – review and editing. **Jessica S. Johnson:** investigation, resources, writing – review and editing. **Casey M. MacDermod:** software, investigation, resources, data curation, writing – review and editing. **Shelby N. Ortiz:** investigation, writing – review and editing. **Emily M. Pisetsky:** investigation, writing – review and editing. **Jennifer P. White:** investigation, resources, data curation, writing – review and editing. **Nadia Micali:** writing – review and editing, supervision, funding acquisition. **Cynthia M. Bulik:** conceptualization, investigation, writing – review and editing, supervision, project administration, funding acquisition. **Laura M. Thornton:** conceptualization, software, investigation, data curation, writing – review and editing, supervision, project administration. **Ya‐Ke Wu:** conceptualization, writing – original draft, writing – review and editing.

## Funding

Data for this study were from the ARFID Genes and Environment study (ARFID‐GEN) R56MH129437 (Bulik/Micali mPIs). Drs. Valdez Aguilar, Ortiz, and Pisetsky were partially supported by the University of North Carolina Suicide Prevention Institute. Ms. Rodriguez received support from T35‐DK007386 from the National Institutes of Health. Dr. Micali reports support from the Novo Nordisk Foundation (Laureate grant award, NNF22OC0071010). Dr. Dinkler reports support from the Swedish Society for Medical Research (SSMF, PG‐22–0478). Dr. Wu reports support from U24DK132715. The content is solely the responsibility of the authors and does not necessarily represent the official views of the National Institutes of Health or the University of North Carolina at Chapel Hill.

## Ethics Statement

This project is a secondary data analysis from the Avoidant Restrictive Food Intake Disorder‐Genes and Environment (ARFID‐GEN). This study was approved by the University of North Carolina Biomedical Institutional Review Board (IRB) Protocol # 22–1524. Informed consent was obtained from all participants. Child assent was also obtained. All methods were carried out in accordance with pertinent guidelines.

## Conflicts of Interest

Dr. Bulik receives royalties from Pearson Education Inc. Dr. Dinkler has received personal fees from Baxter Medical AB and Fresenius Kabi AB outside the submitted work. Other authors declare no competing interests.

## Supporting information


Supporting Information S1

## Data Availability

The data from this study will be available from the National Institute of Mental Health (NIMH) Data Archive (NDA) (https://nda.nih.gov/) under the title Genetic Architecture of Avoidant/Restrictive Food Intake Disorder; ID C4467.
