# Supplementary material for: Associations Between Avoidant/Restrictive Food Intake Disorder Dimensions and Obsessive‐Compulsive Symptomatology
Source: Eur Eat Disord Rev. 2026 Apr 26;34(5):1327–35. doi: 10.1002/erv.70122 (PMC13432419; doi:10.1002/erv.70122)
Supplement: Supplementary file 1 — Supporting Information S1 [file ERV-34-1327-s001.docx]

**Table S1.** Pearson correlations between NIAS and PARDI-AR-Q dimensions with OCI-R subscales, depression, and anxiety.

|  | OCI-R  washing | OCI-R  obsessing | OCI-R  ordering | OCI-R  checking | OCI-R  neutralizing | OCI-R  hoarding | Depression | Anxiety |
| --- | --- | --- | --- | --- | --- | --- | --- | --- |
| NIAS |  |  |  |  |  |  |  |  |
| Picky eating | 0.03 | 0.04* | 0.09* | 0.06* | 0.07* | 0.07* | 0.03 | 0.04* |
| Low appetite | 0.12* | 0.12* | 0.10* | 0.13* | 0.15* | 0.06* | 0.22* | 0.15* |
| Fear | 0.22* | 0.12* | 0.13* | 0.20* | 0.13* | 0.09* | 0.13* | 0.18* |
| PARDI-AR-Q |  |  |  |  |  |  |  |  |
| Sensory-based avoidance | 0.19* | 0.21* | 0.24* | 0.21* | 0.21* | 0.18* | 0.18* | 0.20* |
| Lack of interest | 0.15* | 0.21* | 0.15* | 0.17* | 0.19* | 0.15* | 0.31* | 0.25* |
| Concern about aversive consequences | 0.27* | 0.16* | 0.17* | 0.26* | 0.17* | 0.09* | 0.15* | 0.24* |
| OCI-R total | 0.70* | 0.77* | 0.79* | 0.78* | 0.76* | 0.67* | 0.46* | 0.56* |
| OCI-R washing | 1 | 0.43* | 0.47* | 0.52* | 0.48* | 0.27* | 0.23* | 0.31* |
| OCI-R obesssing |  | 1 | 0.47* | 0.51* | 0.50* | 0.45* | 0.49* | 0.59* |
| OCI-R ordering |  |  | 1 | 0.55* | 0.56* | 0.43* | 0.32* | 0.39* |
| OCI-R checking |  |  |  | 1 | 0.55* | 0.43* | 0.33* | 0.45* |
| OCI-R neutralizing |  |  |  |  | 1 | 0.37* | 0.26* | 0.34* |
| OCI-R hoarding |  |  |  |  |  | 1 | 0.39* | 0.37* |

NIAS = Nine Item Avoidant/Restrictive Food Intake Disorder Screen; PARDI-AR-Q = Pica, ARFID, & Rumination Disorder Inventory Questionnaire; OCI-R = total score from the Obsessive-Compulsive Inventory-Revised; SD = standard deviation; * significant at the p< .05 level

**Table S2.** Mean (SD) for OCI-R subscales for the total sample and by sex. Results and effect sizes from general linear models (GLM) comparing males and females.

| Variables | Total sample  n = 3,120 | Male  n = 274 | Female  n = 2,846 | GLM  Results | Effect size |
| --- | --- | --- | --- | --- | --- |
|  | M (SD) | M (SD) | M (SD) | F (q-value)^a^ | η^2b^ |
| OCI-R |  |  |  |  |  |
| Washing | 2.3 (3.0) | 2.0 (2.9) | 2.3 (3.0) | 4.01 (.09) | 0.001 |
| Obsessing | 4.6 (3.8) | 3.7 (3.7) | 4.7 (3.8) | 18.25 (.0004) | 0.006 |
| Ordering | 4.6 (3.5 | 4.1 (3.4) | 4.7 (3.6) | 7.19 (.018) | 0.002 |
| Checking | 2.7 (2.8) | 2.6 (2.7) | 2.7 (2.8) | 0.92 (.39) | 0.000 |
| Neutralizing | 2.3 (2.9) | 1.7 (2.5) | 2.3 (2.9) | 12.04 (.0004) | 0.004 |
| Hoarding | 4.1 (3.3) | 3.3 (2.9) | 4.2 (3.3) | 19.21 (.0004) | 0.006 |

OCI-R = Obsessive-Compulsive Inventory-Revised; SD = standard deviation. ^a^False Discovery Rate p-values (labeled as q-values) are presented; ^b^Effect size (eta squared, η^2^) is interpreted as follow: Small=0.01, Medium=0.06, Large=0.14

**Table S3.** Results from general linear models examining associations between ARFID dimensions and OCI-R subscales (Model 1) and when accounting for depression (Model 2), anxiety (Model 3), and both depression and anxiety (Model 4). Age and sex were entered into all models as covariates.

|  | Model 1 | | | Model 2 | | | Model 3 | | | Model 4 | | |
| --- | --- | --- | --- | --- | --- | --- | --- | --- | --- | --- | --- | --- |
|  | *B (95% CI)* | F (q-value)^a^ | η_p_^2b^ | *B (95% CI)* | F (q-value)^a^ | η_p_^2b^ | *B (95% CI)* | F (q-value)^a^ | η_p_^2b^ | *B (95% CI)* | F (q-value)^a^ | η_p_^2b^ |
| **Washing** | | | | | | | | | | | | |
| **NIAS** |  |  |  |  |  |  |  |  |  |  |  |  |
| Low appetite | 0.08  (0.05, 0.11) | 34.10 (.0001) | 0.01 | 0.05  (0.02, 0.08) | 12.66  (.0005) | 0.00 | 0.05  (0.03, 0.08) | 15.01  (.0001) | 0.00 | 0.05  (0.02, 0.08) | 13.22  (.0004) | 0.00 |
| Fear | 0.14  (0.12, 0.17) | 153.33  (.0001) | 0.05 | 0.13  (0.11, 0.15) | 141.22  (.0001) | 0.04 | 0.11  (0.09, 0.14) | 112.18  (.0001) | 0.03 | 0.11  (0.09, 0.14) | 111.54  (.0001) | 0.03 |
| **PARDI-AR-Q** |  |  |  |  |  |  |  |  |  |  |  |  |
| Sensory-based avoidance | 0.30  (0.23, 0.36) | 75.27  (.0001) | 0.05 | 0.24  (0.17, 0.30) | 50.44  (.0001) | 0.02 | 0.03  (0.15, 0.28) | 43.33  (.0001) | 0.01 | 0.22  (0.15, 0.28) | 41.88  (.0001) | 0.01 |
| Lack of interest | 0.31  (0.23, 0.40) | 52.46  (.0001) | 0.02 | 0.17  (0.08, 0.26) | 15.39  (.0001) | 0.00 | 0.17  (0.08, 0.25) | 15.29  (.0001) | 0.00 | 0.16  (0.07, 0.24) | 13.03  (.0004) | 0.00 |
| Concern about aversive consequences | 0.45  (0.40, 0.51) | 259.18  (.0001) | 0.08 | 0.41  (0.36, 0.47) | 212.27  (.0001) | 0.06 | 0.36  (0.30, 0.41) | 155.58  (.0001) | 0.05 | 0.36  (0.30, 0.41) | 156.64  (.0001) | 0.05 |
| **Obsessing** | | | | | | | | | | | | |
| **NIAS** |  |  |  |  |  |  |  |  |  |  |  |  |
| Low appetite | 0.08  (0.05, 0.12) | 23.75 (.0001) | 0.01 | -0.01  (-0.03, 0.02) | 0.29  (.605) | 0.00 | 0.01  (-0.02, 0.04) | 0.75  (.407) | 0.00 | 0.00  (-0.04, 0.02) | 0.34  (.580) | 0.00 |
| Fear | 0.10  (0.08, 0.13) | 63.86  (.0001) | 0.02 | 0.06  (0.03, 0.08) | 23.34  (.0001) | 0.01 | 0.03  (0.00, 0.05) | 4.98  (.0312) | 0.00 | 0.02  (0.00, 0.05) | 4.34  (.0425) | 0.00 |
| **PARDI-AR-Q** |  |  |  |  |  |  |  |  |  |  |  |  |
| Sensory-based avoidance | 0.32  (0.24, 0.40) | 59.24  (.0001) | 0.02 | 0.15  (0.09, 0.23) | 18.36  (.0001) | 0.01 | 0.13  (0.06, 0.20) | 13.62  (.0003) | 0.00 | 0.11  (0.04, 0.17) | 9.55  (.0026) | 0.00 |
| Lack of interest | 0.50  (0.40, 0.61) | 91.77  (.0001) | 0.03 | 0.10  (0.00, 0.19) | 3.82  (0.058) | 0.00 | 0.15  (0.06, 0.23) | 10.39  (.0017) | 0.00 | 0.06  (-0.03, 0.15) | 1.77  (.201) | 0.00 |
| Concern about aversive consequences | 0.37  (0.30, 0.44) | 106.26  (.0001) | 0.03 | 0.22  (0.16, 0.29) | 48.52  (.0001) | 0.02 | 0.08  (0.02, 0.14) | 6.12  (.0164) | 0.00 | 0.08  (0.02, 0.14) | 155.10  (.0001) | 0.00 |
| **Ordering** | | | | | | | | | | | | |
| **NIAS** |  |  |  |  |  |  |  |  |  |  |  |  |
| Low appetite | 0.08  (0.04, 0.11) | 21.37 (.0001) | 0.01 | 0.02  (-0.01, 0.05) | 1.72  (.206) | 0.00 | 0.03  (0.00, 0.06) | 4.30  (.041) | 0.00 | 0.02  (-0.01, 0.05) | 1.83  (0.199) | 0.00 |
| Fear | 0.11  (0.08, 0.13) | 64.67  (.0001) | 0.02 | 0.08  (0.05, 0.10) | 35.60  (.0001) | 0.01 | 0.06  (0.03, 0.08) | 19.21  (.0001) | 0.01 | 0.05  (0.03, 0.08) | 18.55  (.0001) | 0.01 |
| **PARDI-AR-Q** |  |  |  |  |  |  |  |  |  |  |  |  |
| Sensory-based avoidance | 0.47  (0.40, 0.55) | 139.56  (.0001) | 0.04 | 0.38  (0.31, 0.46) | 95.46  (.0001) | 0.03 | 0.36  (0.29, 0.43) | 88.85  (.0001) | 0.03 | 0.35  (0.27, 0.42) | 83.44  (.0001) | 0.03 |
| Lack of interest | 0.38  (0.28, 0.48) | 53.08  (.0001) | 0.02 | 0.13  (0.03, 0.23) | 6.23  (.0161) | 0.00 | 0.15  (0.05, 0.25) | 9.33  (.0029) | 0.00 | 0.11  (0.01, 0.21) | 4.44  (.041) | 0.00 |
| Concern about aversive consequences | 0.35  (0.28, 0.42) | 99.36  (.0001) | 0.03 | 0.26  (0.19, 0.33) | 59.45  (.0001) | 0.02 | 0.17  (0.10, 0.24) | 25.30  (.0001) | 0.01 | 0.17  (0.11, 0.24) | 26.38  (.0001) | 0.01 |
| **Checking** | | | | | | | | | | | | |
| **NIAS** |  |  |  |  |  |  |  |  |  |  |  |  |
| Low appetite | 0.08  (0.06, 0.11) | 42.73 (.0001) | 0.01 | 0.04  (0.02, 0.07) | 10.05  (.0019) | 0.00 | 0.04  (0.02, 0.07) | 13.65  (.0003) | 0.00 | 0.04  (0.02, 0.06) | 11.21  (.0011) | 0.00 |
| Fear | 0.13  (0.11, 0.15) | 147.74  (.0001) | 0.04 | 0.10  (0.08, 0.12) | 103.42  (.0001) | 0.03 | 0.08  (0.06, 0.10) | 67.14  (.0001) | 0.02 | 0.08  (0.06, 0.10) | 66.48  (.0001) | 0.02 |
| **PARDI-AR-Q** |  |  |  |  |  |  |  |  |  |  |  |  |
| Sensory-based avoidance | 0.32  (0.26, 0.39) | 104.14  (.0001) | 0.04 | 0.25  (0.19, 0.31) | 64.06  (.0001) | 0.02 | 0.21  (0.16, 0.27) | 53.73  (.0001) | 0.02 | 0.21  (0.15, 0.27) | 51.48  (.0001) | 0.02 |
| Lack of interest | 0.36  (0.28, 0.44) | 78.97  (.0001) | 0.02 | 0.04  (0.08, 0.24) | 15.64  (.0001) | 0.01 | 0.15  (0.08, 0.22) | 15.83  (.0001) | 0.01 | 0.14  (0.06, 0.21) | 12.48  (.0005) | 0.00 |
| Concern about aversive consequences | 0.42  (0.37, 0.47) | 248.46  (.0001) | 0.07 | 0.35  (0.30, 0.41) | 186.04  (.0001) | 0.06 | 0.26  (0.21, 0.31) | 107.46  (.0001) | 0.03 | 0.27  (0.22, 0.32) | 108.69  (.0001) | 0.03 |
| **Neutralizing** | | | | | | | | | | | | |
| **NIAS** |  |  |  |  |  |  |  |  |  |  |  |  |
| Low appetite | 0.09  (0.07, 0.12) | 51.76 (.0001) | 0.01 | 0.06  (0.04, 0.09) | 21.88  (.0001) | 0.01 | 0.07  (0.04, 0.90) | 26.52  (.0001) | 0.01 | 0.06  (0.04, 0.09) | 22.93  (.0001) | 0.01 |
| Fear | 0.08  (0.07, 0.11) | 65.72  (.0001) | 0.02 | 0.07  (0.05, 0.09) | 41.32  (.0001) | 0.01 | 0.05  (0.03, 0.07) | 24.81  (.0001) | 0.01 | 0.05  (0.03, 0.07) | 24.36  (.0001) | 0.01 |
| **PARDI-AR-Q** |  |  |  |  |  |  |  |  |  |  |  |  |
| Sensory-based avoidance | 0.30  (0.24, 0.37) | 86.69  (.0001) | 0.03 | 0.24  (0.18, 0.31) | 57.31  (.0001) | 0.02 | 0.22  (0.16, 0.28) | 50.36  (.0001) | 0.02 | 0.22  (0.16, 0.28) | 48.00  (.0001) | 0.02 |
| Lack of interest | 0.38  (0.29, 0.46) | 82.93  (.0001) | 0.03 | 0.23  (0.15, 0.31) | 29.66  (.0001) | 0.01 | 0.23  (0.15, 0.31) | 31.47  (.0001) | 0.01 | 0.21  (0.13, 0.29) | 26.60  (.0001) | 0.00 |
| Concern about aversive consequences | 0.28  (0.23, 0.34) | 105.02  (.0001) | 0.03 | 0.23  (0.18, 0.29) | 70.95  (.0001) | 0.02 | 0.17  (0.11, 0.22) | 36.80  (.0001) | 0.01 | 0.17  (0.12, 0.22) | 37.50  (.0001) | 0.01 |
| **Hoarding** | | | | | | | | | | | | |
| **NIAS** |  |  |  |  |  |  |  |  |  |  |  |  |
| Low appetite | 0.04  (0.01, 0.07) | 5.63  (.0218) | 0.00 | -0.03  (-0.06, 0.00) | 4.51  (.0396) | 0.00 | 0.00  (-0.03, 0.02) | 0.07  (.793) | 0.00 | -0.03  (-0.06, 0.00) | 4.61  (.0374) | 0.00 |
| Fear | 0.07  (0.05, 0.09) | 32.37  (.0001) | 0.01 | 0.03  (0.01, 0.06) | 8.52  (.0050) | 0.00 | 0.02  (0.00, 0.05) | 3.85  (.057) | 0.00 | 0.02  (0.00, 0.04) | 3.17  (.080) | 0.00 |
| **PARDI-AR-Q** |  |  |  |  |  |  |  |  |  |  |  |  |
| Sensory-based avoidance | 0.31  (0.26, 0.44) | 53.32  (.0001) | 0.02 | 0.20  (0.13, 0.27) | 32.44  (.0001) | 0.01 | 0.21  (0.14, 0.28) | 34.60  (.0001) | 0.01 | 0.18  (0.11, 0.25) | 26.88  (.0001) | 0.01 |
| Lack of interest | 0.35  (0.29, 0.46) | 82.93  (.0001) | 0.02 | 0.05  (-0.04, 0.14) | 1.16  (.303) | 0.00 | 0.15  (0.06, 0.24) | 10.20  (.0018) | 0.00 | 0.04  (-0.05, 0.13) | 0.63  (.446) | 0.00 |
| Concern about aversive consequences | 0.18  (0.11, 0.24) | 28.74  (.0001) | 0.01 | 0.07  (0.01, 0.13) | 4.87  (.0333) | 0.00 | 0.01  (-0.06, 0.07) | 0.04  (.852) | 0.00 | 0.01  (-0.05, 0.07) | 0.17  (.696) | 0.00 |

*B*  = parameter estimates; NIAS = Nine Item Avoidant/Restrictive Food Intake Disorder Screen; PARDI-AR-Q = the Pica, ARFID, & Rumination Disorder Inventory Questionnaire); ^a^False Discovery Rate p-values (labeled as q-values) are presented; ^b^Effect size (partial eta squared; η_p_^2^) is interpreted as follow: Small=0.01, Medium=0.06, Large=0.14
